# Bilateral coronary artery fistulas with progressive aortic regurgitation presenting as persistent atrial fibrillation, acute heart failure and pulmonary hypertension: a case report

**DOI:** 10.3389/fcvm.2026.1776492

**Published:** 2026-04-28

**Authors:** Jixia Feng, Xiaorong Xia, Jiafu Li, Xuefeng Wang

**Affiliations:** 1 Department of Cardiology, Hejiang People's Hospital, Luzhou, Sichuan, China; 2 Department of Cardiology, The Affiliated Hospital, Southwest Medical University, Luzhou, Sichuan, China; 3 Department of Ultrasound, The Affiliated Hospital, Southwest Medical University, Luzhou, Sichuan, China

**Keywords:** aortic regurgitation, atrial fibrillation, coronary artery fistula, heart failure, pulmonary hypertension

## Abstract

Coronary artery fistula (CAF) represents a rare cardiovascular anomaly, defined by an abnormal connection between one or more coronary arteries and the cardiac chambers or other intrathoracic vessels. Most CAFs are congenital, single, small, and asymptomatic, often discovered incidentally. Nonetheless, some cases possess potential clinical implications, including arrhythmia, congestive heart failure, cardiomyopathy and pulmonary hypertension. Furthermore, CAFs may coexist with other congenital cardiac anomalies, posing significant diagnostic and management challenges. This case report describes a 50-year-old male patient with progressive left atrial dilation and aortic regurgitation, who subsequently developed persistent atrial fibrillation, acute heart failure, and pulmonary hypertension. Incidentally, contrast-enhanced computed tomography and subsequent coronary angiography confirmed the presence of bilateral coronary-to-pulmonary artery fistulas. Stress myocardial perfusion scintigraphy demonstrated significant myocardial ischemia in the territory of the left anterior descending coronary artery. Right heart catheterization indicated combined post-and precapillary pulmonary hypertension and a moderate left-to-right shunt. In addition to receiving guideline-directed medical therapy, the patient underwent atrial fibrillation catheter ablation and simultaneous transcatheter CAFs closure. During a one-year follow-up period, the patient maintained sinus rhythm and reported overall well-being. Echocardiography and cardiac magnetic resonance imaging revealed significant recovery from adverse cardiac remodeling. This case underscores the significance of comprehensive cardiac evaluations in patients with atrial fibrillation and/or heart failure, as atypical findings can substantially influence diagnoses, management, and outcomes. Furthermore, given the complex interrelationship between CAFs, aortic regurgitation, atrial fibrillation, and heart failure, this report highlights the necessity for individualized treatment strategies that integrate pharmacological, interventional, and surgical approaches.

## Introduction

Coronary artery fistula (CAF) is a rare cardiovascular anomaly, characterized by an abnormal connection between one or more coronary arteries and the cardiac chambers or other intrathoracic vessels (1). Due to variations in study populations and designs, the prevalence of CAF ranges from 0.06% to 0.91% (2). The majority of CAFs are congenital in nature; however, increasing CAFs arising from iatrogenic (e.g., pacemaker implantation, percutaneous coronary artery intervention, endomyocardial biopsy and cardiac surgery), traumatic or infectious causes have been reported (2–4). While most CAFs are single, small, and asymptomatic, often discovered incidentally during coronary angiography or other noninvasive cardiac imaging, some cases carry potential clinical implications, including arrhythmia, congestive heart failure, cardiomyopathy, pulmonary hypertension, and even sudden cardiac death (5).

Currently, there is a lack of high-quality evidence to guide the management of CAFs. Most consensus approaches are derived from clinical experience, taking into account symptoms, CAF morphologies, resultant complications, and procedural factors to determine the indication and optimal method for closing CAFs (5). For symptomatic large or medium CAFs, surgical or transcatheter closure is recommended in the presence of ischemia, arrhythmia, endarteritis, vessel rupture, cardiac chamber enlargement, and ventricular dysfunction (1, 5). However, the optimal management strategy for small and/or asymptomatic CAFs remains unclear. Furthermore, CAFs can occasionally coexist with other congenital heart diseases or cardiomyopathies, such as atrial septal defect, ventricular septal defect, patent ductus arteriosus, pulmonary atresia with intact ventricular septum, tetralogy of Fallot, hypertrophic cardiomyopathy, dilated cardiomyopathy, arrhythmogenic right ventricular cardiomyopathy and left ventricular noncompaction (6–10). Involvement of the aorta or aortic valves, including conditions such as aortic root aneurysm, bicuspid aortic valve stenosis, bicuspid aortic valve insufficiency, and sinus of Valsalva aneurysms, has been infrequently documented, adding complexity to the diagnosis, assessment, management, and prognosis of CAFs (11–15).

In this report, we present a case involving bilateral coronary-to-pulmonary artery fistulas and progressive aortic regurgitation (AR), manifesting as persistent atrial fibrillation (AF), acute heart failure, and pulmonary hypertension. The patient underwent successful AF catheter ablation and concomitant transcatheter closure of the CAFs, resulting in a favorable clinical outcome. This case underscores the importance of comprehensive cardiac evaluations and the implementation of tailored treatment strategies for patients with CAFs and other congenital cardiac anomalies.

## Case presentation

A 50-year-old male government employee was admitted to our department in November 2024, presenting with rapidly progressive dyspnea and bilateral lower limb edema. The patient reported the onset of exertional dyspnea two weeks prior to admission, which had progressively deteriorated to severe heart failure, classified as New York Heart Association (NYHA) class III. His medical history is notable for an uneventful cerebral aneurysm coil closure six years prior and a one-year history of hypertension, with no reported family history of hereditary disease.

On physical examination, the patient exhibited an atrial fibrillation rhythm, with a heart rate of 98 beats per minute and a pulse rate of 85 beats per minute. The patient's body mass index was calculated at 28.7 Kg/m^2^, and the blood pressure was measured at 120/76 mm Hg. Additional findings included bilateral moist rales, cardiomegaly, a mild diastolic heart murmur (most prominent at the second intercostal space along the left parasternal border) and bilateral lower limb edema.

### Diagnostic assessment and therapeutic intervention

Laboratory investigations, including complete blood cell counts, renal function tests, electrolyte levels, lipid profiles, arterial blood gas analysis, D-dimer, and high-sensitivity troponin T level, were largely unremarkable, with the exception of a significantly elevated alanine aminotransferase level (ALT: 154.9 U/L; normal range: 9–50 U/L), aspartate aminotransferase level (AST: 81.4 U/L; normal range:15–40 U/L), and N-terminal pro-B-type natriuretic peptide level (NT-pro BNP: 2,692 pg/mL; normal range: ≤300 pg/mL).

**Figure 1:**
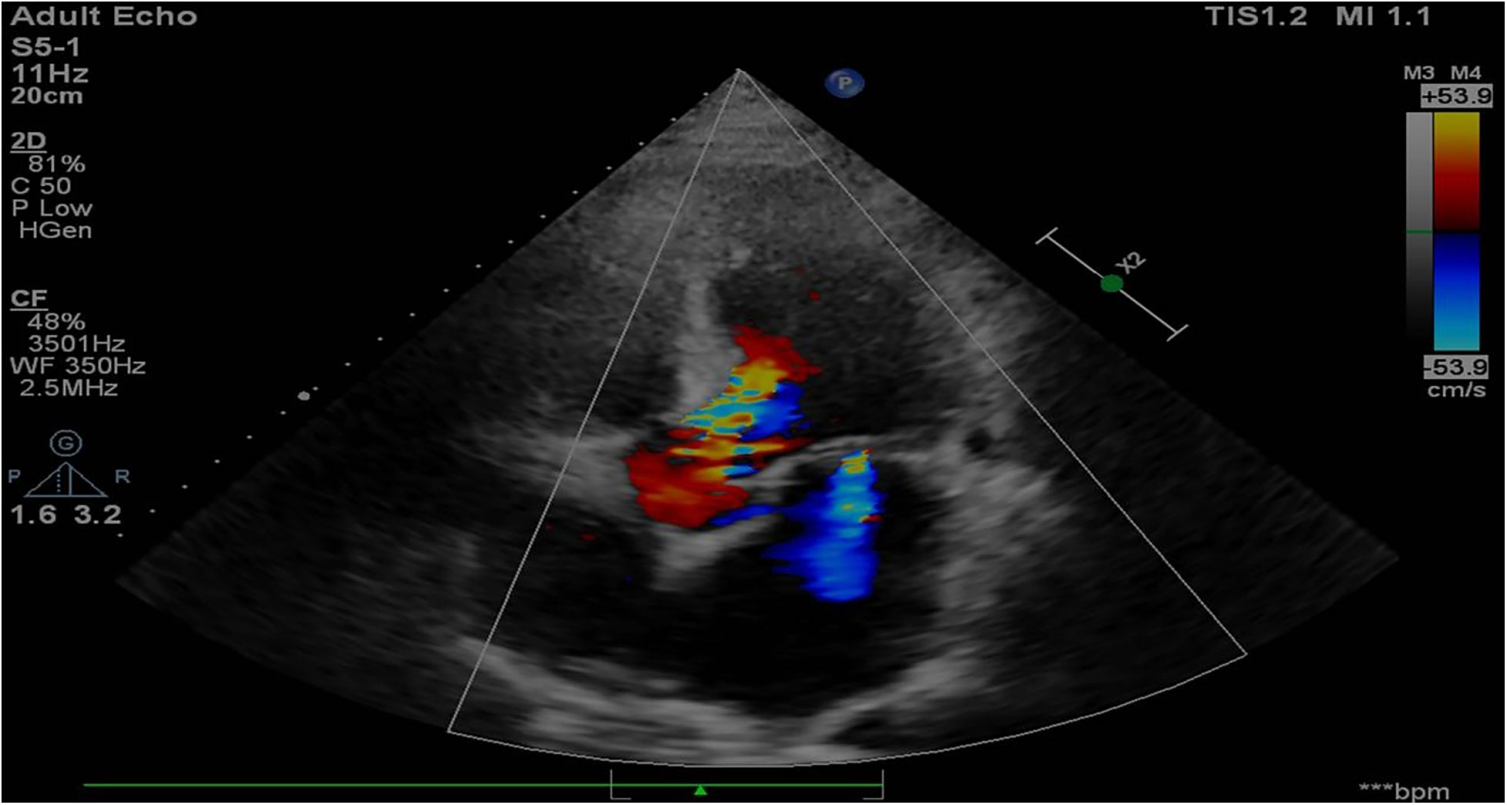
Echocardiogram conducted at the time of admission showing global cardiac dilation and moderate aortic regurgitation.

The patient's other clinical data from recent years is summarized in Table 1. Notably, despite being asymptomatic prior to the current admission, there was a gradual progression of left atrial dilation. More significantly, the patient's mild AR advanced to moderate AR, and persistent atrial fibrillation developed two months before the current admission. Additionally, an echocardiogram conducted at the time of admission revealed global cardiac dilation, moderate AR, mild mitral and tricuspid regurgitation, mild pericardial effusion, pulmonary hypertension (estimated pulmonary artery pressure: 41 mm Hg), and a mildly reduced left ventricular ejection fraction (LVEF: 49%) (Figure 1). A chest computed tomography scan indicated cardiomegaly, mild pericardial effusion, pulmonary artery dilation, and bilateral pleural effusions (Supplementary Figure S1A).

**Table 1:** Clinical data from recent years.

Examination Date		2022.10	2023.09	2024.09	2024.11	2025.12
Department		Health Management Center	Health Management Center	Health Management Center	Inpatient Department	Outpatient Clinics
Asymptomatic/Symptoms		Asymptomatic	Asymptomatic	Asymptomatic	Dyspnea, lower limb edema	Asymptomatic
Electrocardiogram		Sinus rhythm	Sinus rhythm	Atrial fibrillation	Atrial fibrillation	Sinus rhythm
Echocardiography	Left atrium, mm	34	37	39	49	32
	Left ventricle, mm	Normal	Normal	53	62	58
	Aorta, mm	36	40	38	38	37
	Aortic regurgitation	Mild	Mild	Moderate	Moderate	Moderate
	Mitral regurgitation	None	None	Mild	Mild	None
	Tricuspid regurgitation	None	None	Mild	Mild	None
	Left ventricular ejection fraction, %	Normal	Normal	58%	49%	58%
	Others	-	-	Arrhythmia	Right atrium: 62 mm, Right ventricle: 27 mm, Mild pericardial effusion, Pulmonary hypertension, Arrhythmia	-

**Figure 2:**
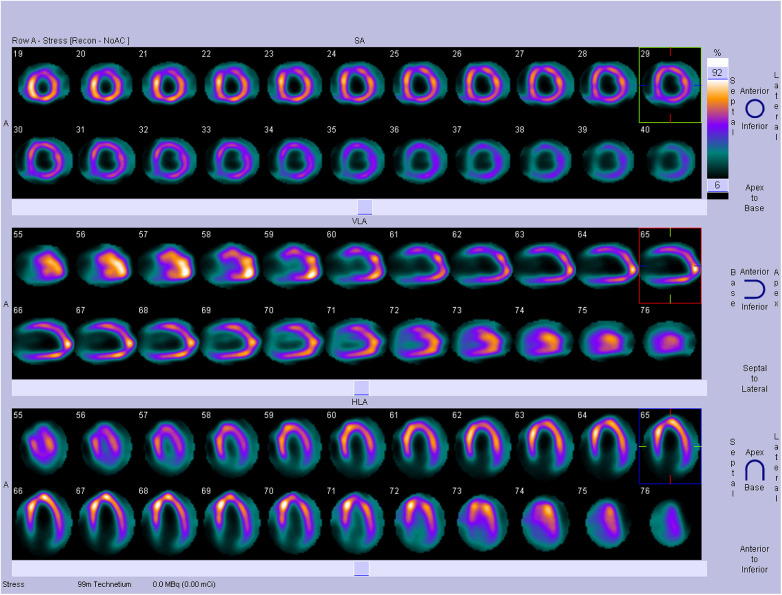
Single-photon emission computed tomography stress myocardial perfusion scintigraphy showing substantial hypoperfusion in the left ventricular anterior wall and anterior septum wall.

**Figure 3:**
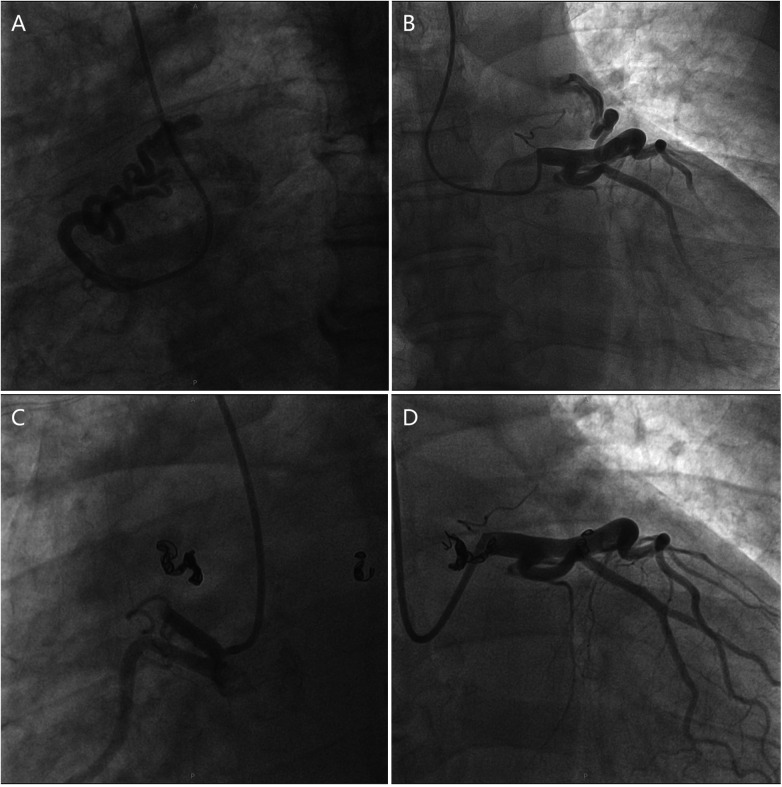
**(A,B)** Invasive coronary angiogram showing bilateral coronary-to-pulmonary artery fistulas. **(C,D)** Post-coronary artery fistula closure coronary angiogram showing successful closure of coronary artery fistulas.

The therapeutic regimen included intravenous torsemide, spironolactone, rivaroxaban, dapagliflozin, and sacubitril/valsartan, to which the patient responded favorably. Given the significant role that persistent AF and moderate AR played in the onset and rapid progression of adverse cardiac remodeling and heart failure, surgical AR correction and concomitant Maze procedure were proposed. However, the patient declined open-heart surgery and consented only to AF catheter ablation. Transesophageal echocardiography showed dilation of the left atrium and ruled out the presence of left atrial appendage thrombus. Notably, pre-ablation computed tomography pulmonary venography revealed some anomalous vessels around the main pulmonary artery, indicating suspicious CAFs (Supplementary Figure S2). Further single-photon emission computed tomography stress myocardial perfusion scintigraphy demonstrated significant hypoperfusion in the left ventricular anterior wall and anterior septum, corresponding to the territory supplied by the left anterior descending coronary artery (LAD) (Figure 2). Subsequent invasive coronary angiography confirmed the presence of bilateral coronary-to-pulmonary artery fistulas (Figures 3A, B; Supplementary Videos S1, S2). Right heart catheterization indicated combined post- and pre-capillary pulmonary hypertension, with a pulmonary artery pressure of 58/34 (mean 42) mm Hg, a pulmonary vascular resistance of 3.2 Wood Units and a Qp:Qs ratio of 1.5 (Supplementary Table S1). Considering the interplay between silent myocardial ischemia, volume overload, atrial fibrillation, heart failure, and pulmonary hypertension, both AF catheter ablation and concomitant transcatheter closure of CAFs were successfully performed (Figures 3C, D; Supplementary Videos S3, S4). Postoperatively, the patient's physical activity showed further improvement, leading to discharge four days later with a medication regimen including torsemide, spironolactone, bisoprolol, rivaroxaban, dapagliflozin, and sacubitril/valsartan.

### Follow-up and outcomes

During a one-year follow-up period, the patient successfully maintained sinus rhythm and reported an overall sense of well-being, despite suboptimal adherence to the prescribed medication regimen, with no recurrence of dyspnea. An echocardiogram and chest computed tomography conducted one year after the AF catheter ablation and CAF closure indicated normalization of the patient's right heart, left atrium, LVEF, and estimated systolic pulmonary pressure. Furthermore, mild mitral and tricuspid regurgitation, as well as pericardial effusion resolved, and there was a significant improvement in the dilation of the left ventricle, while moderate aortic regurgitation remained stable (Table 1 and Supplementary Figure S1B). Furthermore, cardiac magnetic resonance imaging (CMRI) corroborated the echocardiographic findings, showing a similar degree of left ventricular dilation, and mild myocardial scarring in the mid-layer of the basal ventricular septum (Supplementary Figure S3).

## Discussion

Coronary artery fistula (CAF) is a rare coronary anomaly, characterized by an abnormal connection between one or more coronary arteries and the cardiac chambers or other intrathoracic vessels without an interposed capillary bed (1). The prevalence of CAF varies between 0.06% and 0.91%, largely due to differences in study populations and methodologies (2). The majority of CAFs are congenital in nature; however, there has been an increase in cases attributed to iatrogenic causes, such as pacemaker implantation, percutaneous coronary interventions, endomyocardial biopsies, and cardiac surgeries, as well as traumatic or infectious etiologies (2–4). Congenital fistulas that terminate in a cardiac chamber are referred to as coronary cameral fistulas, whereas those that drain into an intrathoracic vessel are termed coronary arteriovenous fistulas (16). These anomalies arise from the persistence of sinusoids that fail to regress during early embryonic development and the retention of primitive connections between coronary arteries and other intrathoracic vessels, respectively (10). Although most CAFs are typically single, small, and asymptomatic—often discovered incidentally via coronary angiography or other non-invasive cardiac imaging modalities—some cases may have significant clinical implications. These can include silent myocardial ischemia, volume overload, and subsequent complications such as arrhythmias, congestive heart failure, cardiomyopathy, pulmonary hypertension, coronary aneurysm rupture, and even sudden cardiac death (2, 8, 17–19). For patients with CAFs who qualify for intervention, both transcatheter and surgical closure techniques have demonstrated favorable outcomes (20–22).

Transthoracic echocardiography is the preferred modality for evaluating cardiac structure and function due to its non-invasive nature and cost-effectiveness. The presence of an abnormal jet or an aberrant vessel with blood flow on echocardiography is highly indicative of CAFs (23). A previous echocardiographic study demonstrated a high diagnostic accuracy for CAFs (24). However, our recent investigation found that echocardiography correctly diagnosed only 9% of patients with CAF, with the majority of fistulas exceeding 4.5 mm in size (2). In contrast to echocardiography, coronary computed tomography angiography and CMRI are not limited by acoustic windows and offer superior spatial resolution, thereby providing more comprehensive anatomical details of fistulas, which are crucial for the effective management of CAFs (25). Furthermore, CMRI with myocardial perfusion imaging and/or late gadolinium enhancement possess unique tissue characterization capabilities, enabling precise assessment of myocardial ischemia and fibrosis *in vivo*. Despite these technological advancements, invasive coronary angiography remains the gold standard diagnostic imaging modality, and allows for simultaneous transcatheter closure of CAFs with occlusive coils or other devices (10). In our case, neither transthoracic nor transesophageal echocardiography successfully visualized the patient's CAFs. However, computed tomography pulmonary venography conducted prior to atrial fibrillation catheter ablation successfully identified the fistulas. Subsequent invasive coronary angiography confirmed the diagnosis of bilateral coronary-to-pulmonary artery fistulas. Additionally, CMRI revealed myocardial fibrosis in the mid-layer of the basal ventricular septum. Thus, echocardiography appears to have limited sensitivity in detecting CAFs, particularly those of small or medium size. Comprehensive imaging approaches, including echocardiography, contrast-enhanced computed tomography, CMRI, and invasive coronary angiography, are crucial for the accurate diagnosis and assessment of CAFs.

The primary pathophysiological mechanisms associated with CAFs are the coronary steal phenomenon and volume overload (10). Over time, CAFs may enlarge, particularly in pediatric and young adult populations, potentially leading to the progression of myocardial ischemia and left-to-right extracardiac shunt (26), and subsequently various complications, such as arrhythmias, congestive heart failure, cardiomyopathy, pulmonary hypertension, and even sudden cardiac death (5, 17). In our case study, the patient had never experienced angina pectoris, and his electrocardiogram showed no ischemic changes, with all coronary arteries remaining patent. However, myocardial stress perfusion scintigraphy revealed significant myocardial hypoperfusion in the LAD territory, which may have important clinical implications. Consequently, it is prudent to assess silent myocardial ischemia in asymptomatic patients with CAFs to inform appropriate management strategies. Furthermore, in recent years, a series of echocardiograms conducted during health check-ups have revealed a progressive worsening of left atrial dilation and aortic regurgitation in the patient. Despite his relatively young age and absence of other cardiovascular disease risk factors at that time, it is plausible that longstanding silent myocardial ischemia, along with concomitant volume overload due to CAFs and AR, has contributed to adverse left atrial remodeling and subsequent atrial fibrillation. Therefore, despite the inherent challenges, early diagnosis, accurate assessment, and prompt closure of CAFs have the potential to mitigate these pathophysiological processes and lead to more favorable clinical outcomes.

Currently, there is a lack of high-quality evidence to guide the management of CAFs. Most consensus-based approaches are derived from clinical experience, taking into account symptoms, CAF morphologies, and procedural factors to determine the indication and optimal method for closing CAFs (5). For symptomatic large or medium CAFs, surgical or transcatheter closure is recommended in the presence of ischemia, arrhythmia, endarteritis, vessel rupture, cardiac chamber enlargement, and ventricular dysfunction (1, 5). In contrast, small CAFs are generally considered to be of minimal clinical significance and can be monitored without any intervention (5). Prior to admission, the present patient was asymptomatic, and his CAFs were not large. However, the sequelae of his CAFs—longstanding silent myocardial ischemia, moderate left-to-right extracardiac shunt, progressive left atrial dilation, persistent atrial fibrillation, and ultimately adverse cardiac remodeling and dysfunction, pulmonary hypertension, and myocardial scarring—were severe. This suggests that evaluating CAFs solely based on their symptoms, CAF sizes, and resultant complications is insufficient, as it may delay treatment and lead to serious consequences. Our recent angiographic study revealed that the majority of CAFs, although small, were symptomatic (2). Furthermore, arrhythmias, adverse cardiac remodeling, and left ventricular dysfunction were not uncommon (2). Our unpublished study further demonstrated that the morphologies of CAFs (including their numbers, diameters, origins, and terminations) appeared unrelated to cardiac phenotypes such as arrhythmia, adverse cardiac remodeling, left ventricular dysfunction, and pulmonary hypertension, indicating significant heterogeneity among CAFs. In the context of small and/or asymptomatic CAFs, it is essential to include not only the morphological characteristics of CAFs but also the extent of left-to-right shunts, the presence of silent myocardial ischemia (optimally evaluated through myocardial stress perfusion scintigraphy), unexplained arrhythmias, cardiac chamber dilation, left ventricular dysfunction, and pulmonary hypertension in the risk stratification process. Nonetheless, further research is warranted to solve these considerations comprehensively.

Notably, CAFs may coexist with other congenital cardiac anomalies (6–10). Involvement of the aorta or aortic valves, including conditions such as aortic root aneurysm, bicuspid aortic valve stenosis, bicuspid aortic valve insufficiency, and sinus of Valsalva aneurysms, has been infrequently documented in patients with CAFs (11–15). The coexistence of bilateral CAFs, organic AR, and AF is rare, posing considerable challenges in both diagnosis and management. Organic AR, AF, and subsequent heart failure may obscure the symptoms of CAFs, potentially leading to missed diagnoses. Conversely, given that the majority of the aortic valve's blood supply is derived from the coronary arteries, the coronary steal phenomenon and volume overload caused by CAFs may promote the progression of AR, thereby complicating therapeutic strategies (27). Notably, the patient developed severe congestive heart failure, global cardiac dilation and dysfunction, and pulmonary hypertension two months following the initial diagnosis of AF and moderate AR. This rapid progression underscores the significant impact of persistent AF and moderate AR on the onset and rapid deterioration of adverse cardiac remodeling and heart failure. In this scenario, surgical intervention involving AR correction, CAF closure, and AF ablation should be considered. However, the patient declined.

Both surgical and transcatheter approaches to CAF closure have been associated with favorable outcomes (20, 21). Given the severity of the patient's condition and the anatomical characteristics of the CAFs, transcatheter closure of the CAFs is technically feasible, minimally invasive, and should be prioritized in cases where the patient declines surgical intervention. Considering the interplay among silent myocardial ischemia, volume overload, atrial fibrillation, heart failure, and pulmonary hypertension, both transcatheter closure of CAFs and concomitant AF catheter ablation were performed, resulting in a favorable outcome.

Additionally, although the patient exhibited a moderate left-to-right extracardiac shunt (Qp:Qs = 1.5), he had not previously reported any symptoms, and earlier echocardiographic evaluations did not reveal the presence of pulmonary hypertension. At the time of admission, the patient's post- and pre-capillary pulmonary hypertension was primarily attributed to congestive heart failure. Therefore, pulmonary hypertension medications were not indicated.

Interestingly, the patient's CAF directly originating from the right coronary sinus is, in fact, a fistula between the right conus artery and the main pulmonary artery (28). The right conus artery, which arises directly from the right coronary sinus, is also referred to as the third coronary artery or accessory coronary artery. This anatomical variation is relatively common, occurring in approximately 50% of the general population (29). CAFs that originate from the third coronary artery display hemodynamic characteristics similar to those observed in patent ductus arteriosus, without the occurrence of the coronary steal phenomenon. Notably, this type of CAF may be overlooked during coronary angiography due to “the proficiency of current intubation techniques”. Therefore, increased attention should be directed towards identifying these CAFs, particularly in patients with left coronary artery fistulas.

Lastly, recent investigations have also highlighted the significant role of genetic abnormalities in formation of CAFs (14, 30–32). In light of our patient's concurrent presence of bilateral CAFs, early-onset cerebral aneurysm, and organic AR—all likely congenital anomalies—this constellation strongly indicates an underlying genetic defect. Regrettably, genetic testing was declined.

## Conclusions

CAF represents a rare cardiovascular anomaly. While the majority of CAFs are congenital, single, small, and asymptomatic—often identified incidentally—some cases possess the potential to induce clinical complications such as arrhythmias, congestive heart failure, cardiomyopathy, and pulmonary hypertension. Furthermore, CAFs may occasionally coexist with other congenital cardiac anomalies, thereby posing significant diagnostic and therapeutic challenges. The utilization of multimodal imaging techniques, including echocardiography, computed tomography, cardiac magnetic resonance imaging, invasive coronary artery angiography, and myocardial stress perfusion scintigraphy, is essential for the precise diagnosis and comprehensive evaluation of CAFs. Tailored treatment approaches, encompassing pharmacological, interventional, and surgical strategies, should be considered for patients presenting with CAF and concurrent cardiovascular anomalies.

## Patient perspective

From the patient's point of view, the interventional therapy was accepted. During a one-year follow-up period, the patient successfully maintained sinus rhythm and reported an overall sense of well-being, with no recurrence of dyspnea.

## Data Availability

The original contributions presented in the study are included in the article/Supplementary Material, further inquiries can be directed to the corresponding authors.

## Supplementary material

Supplementary Figure S1 **(A)** A chest computed tomography scan conducted at the time of admission showing cardiomegaly and pleural effusions. **(B)** A chest computed tomography scan performed one year after coronary artery fistula closure and atrial fibrillation catheter ablation demonstrating significant improvement in the patient's cardiac dilation.

Supplementary Figure S2 Computed tomography pulmonary venography showing some anomalous vessels around the main pulmonary artery.

Supplementary Figure S3 Cardiac magnetic resonance imaging showing mild myocardial scarring in the mid-layer of the basal ventricular septum segment.

Supplementary Videos S1–S4 Pre- and post-coronary artery fistula closure coronary angiogram videos.
