# Supplementary figures and images for: Bilateral coronary artery fistulas with progressive aortic regurgitation presenting as persistent atrial fibrillation, acute heart failure and pulmonary hypertension: a case report
Source: Front Cardiovasc Med. 2026 Apr 28;13:1776492. doi: 10.3389/fcvm.2026.1776492 (PMC13161127; doi:10.3389/fcvm.2026.1776492)

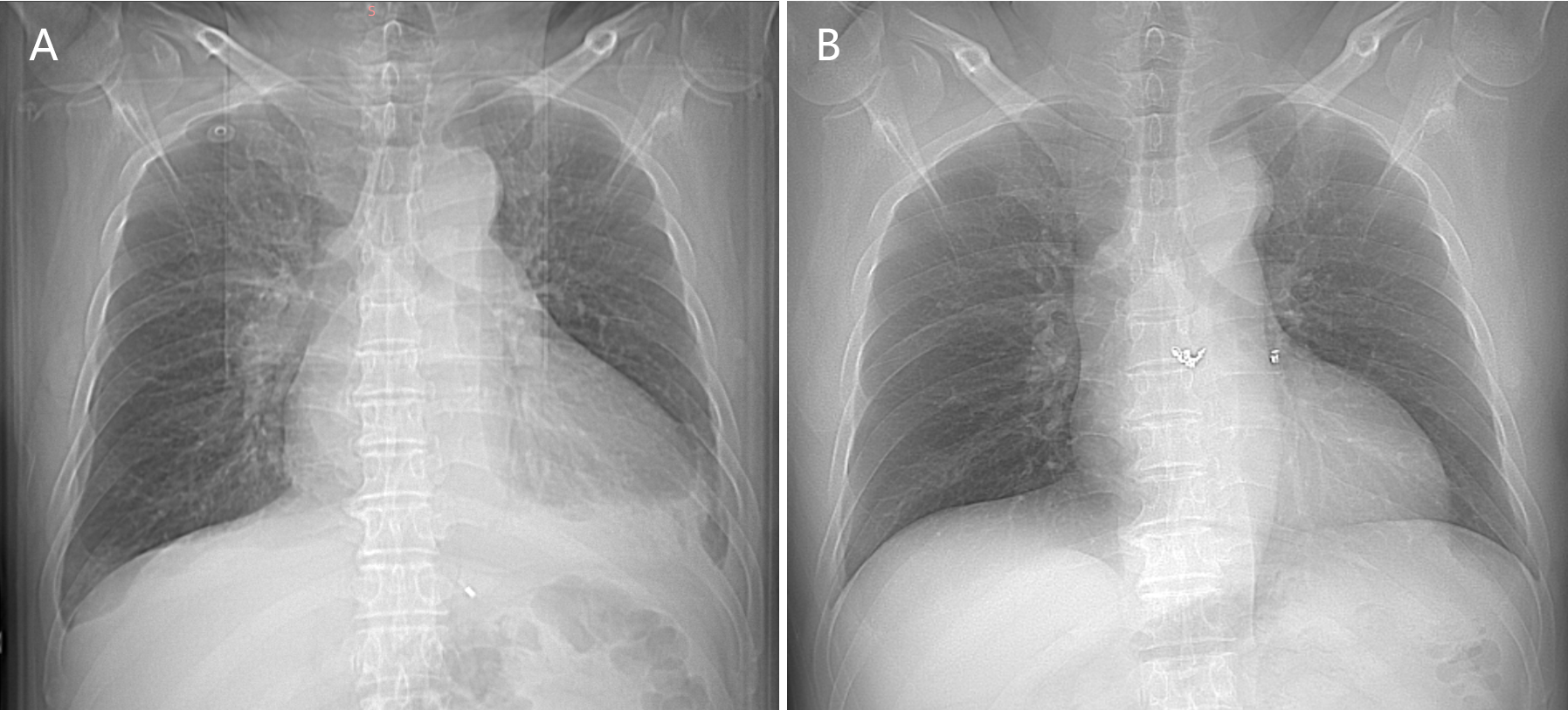

Supplement: Supplementary Figure S1 — (A) A chest computed tomography scan conducted at the time of admission showing cardiomegaly and pleural effusions. (B) A chest computed tomography scan performed one year after coronary artery fistula closure and atrial fibrillation catheter ablation demonstrating significant improvement in the patient's cardiac dilation. [file Image1.tif]

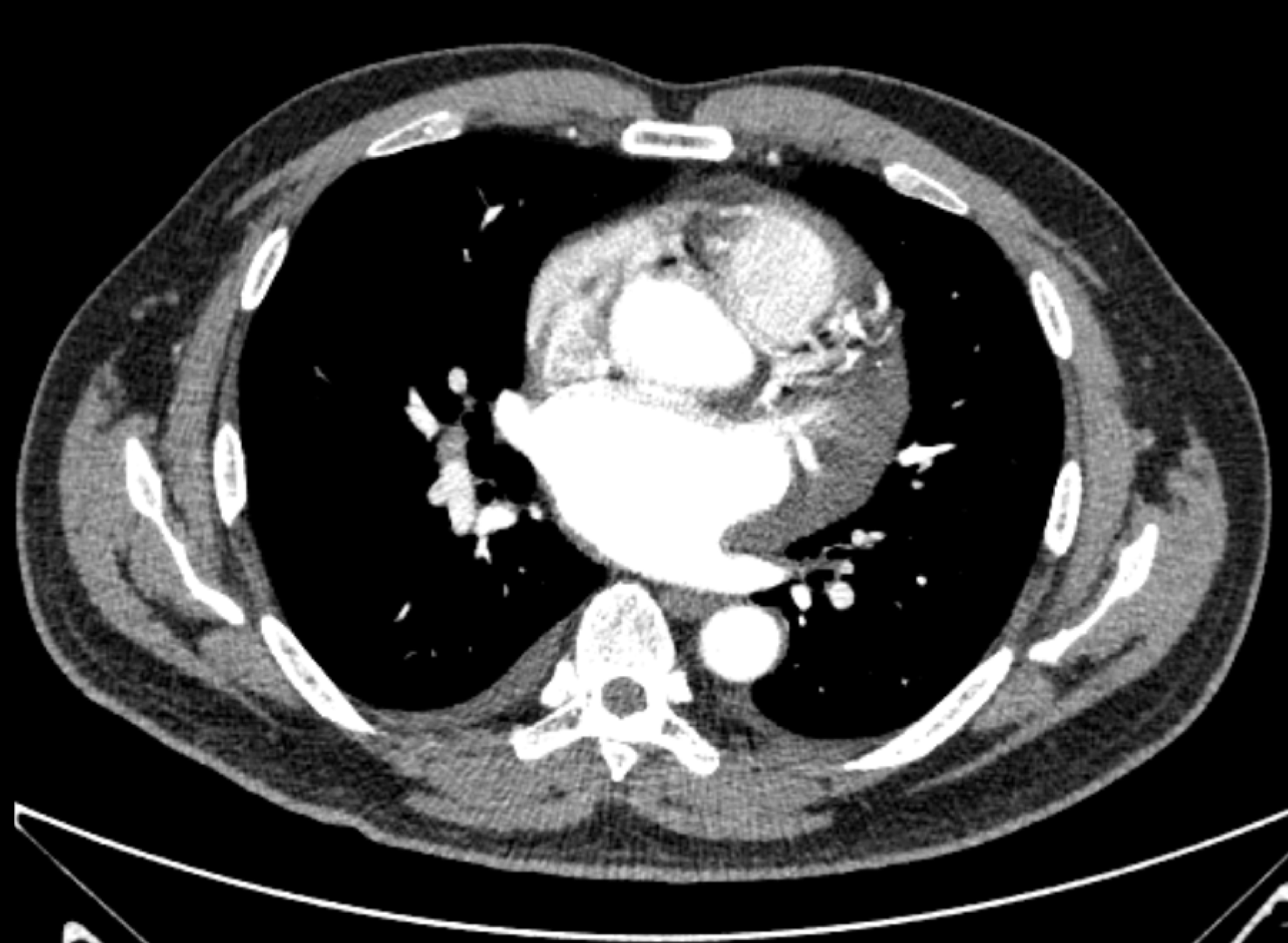

Supplement: Supplementary Figure S2 — Computed tomography pulmonary venography showing some anomalous vessels around the main pulmonary artery. [file Image2.tiff]

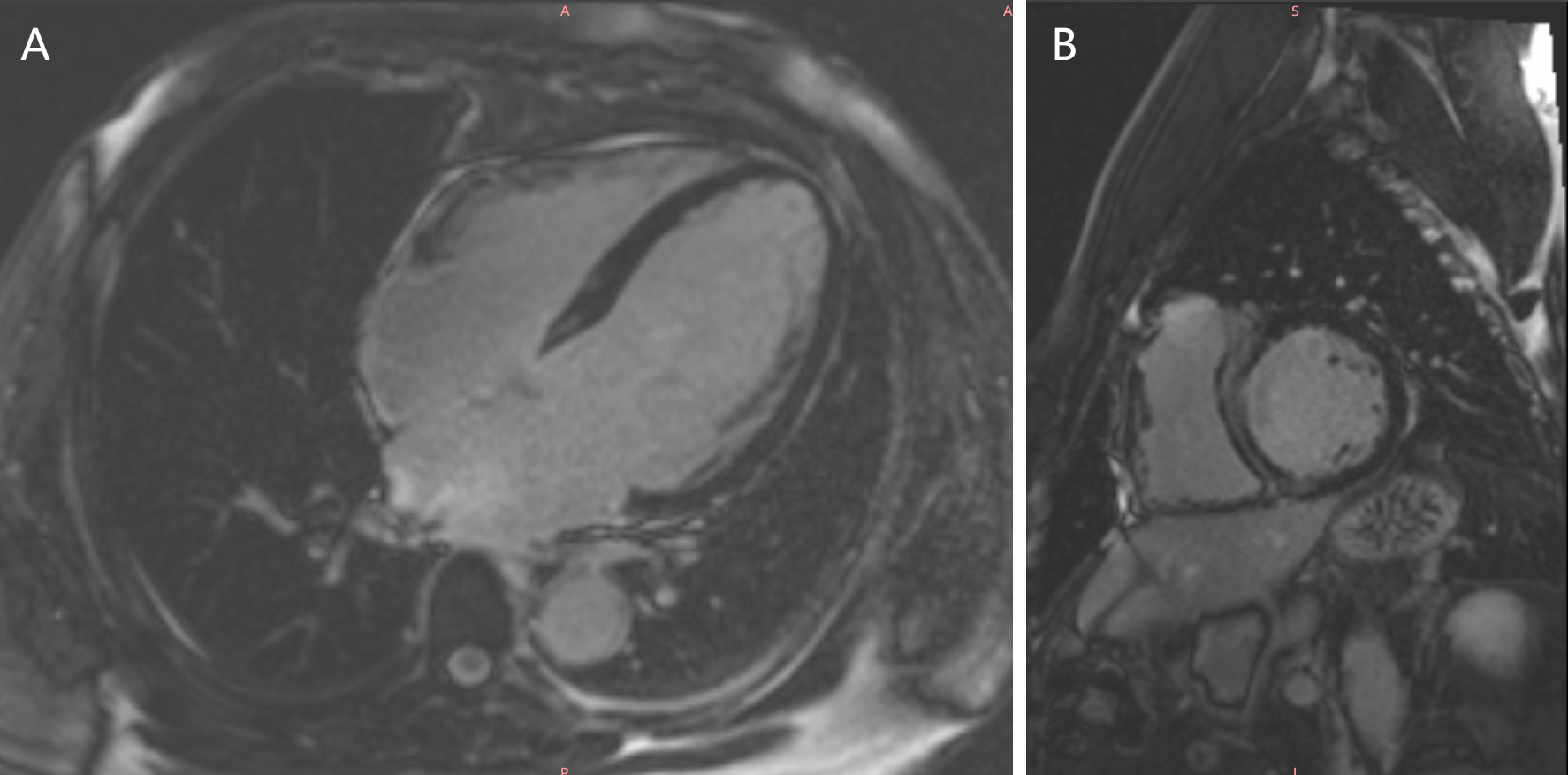

Supplement: Supplementary Figure S3 — Cardiac magnetic resonance imaging showing mild myocardial scarring in the mid-layer of the basal ventricular septum segment. [file Image3.tif]
